# Evaluation of the HAL® lumbar type exoskeleton in long-term care: protocol for a mixed-methods feasibility study

**DOI:** 10.3389/fdgth.2025.1591866

**Published:** 2025-08-18

**Authors:** Sebastian Hofstetter, Pascal Müller, Dominik Behr, Suenye Thal, Motoaki Komiya, Stefan Dowiasch, Patrick Jahn

**Affiliations:** ^1^ Department of Internal Medicine, Faculty of Medicine, University Medicine Halle (Saale), Health Service Research Working Group | Acute Care, Martin Luther University Halle-Wittenberg, Halle (Saale), Germany; ^2^ Cyberdyne Care Robotics GmbH Hunscheidtstr, Bochum, Germany; ^3^ Department of Nursing, Midwifery and Therapeutic Sciences, Medical Devices and Technologies, Bochum University of Applied Sciences, Health Campus, Bochum, Germany

**Keywords:** exoskeleton, long-term care, caregivers, usability, occupational health, assistive technology

## Abstract

**Background:**

Lower back pain (LBP) is one of the most common occupational health issues among healthcare professionals, particularly in long-term care settings. The HAL® Lumbar Type Exoskeleton is a wearable assistive technology designed to reduce strain on the lower back during physically demanding care activities. However, evidence regarding its feasibility, usability, and acceptance in real-world long-term care settings remains limited.

**Objective:**

This study aims to evaluate the feasibility, usability, and user acceptance of the HAL® Lumbar Type Exoskeleton in long-term care facilities. Specifically, the study assesses whether the exoskeleton can reduce self-reported lower back pain and improve the ergonomic conditions for caregivers.

**Methods:**

This is a non-randomized, exploratory interventional feasibility study using a mixed-methods design. A total of 30 caregivers from two long-term care facilities will participate in a 90-day intervention. The exoskeleton will be integrated into daily care routines, and caregivers will undergo training on its proper use.

**Quantitative measures include:**

Visual Analog Scale (VAS) for pain assessment before and after using the exoskeleton. Oswestry Disability Index (ODI) and Short-Form Health Survey (SF-8) at baseline (T1), mid-study (T2), and post-intervention (T3). Qualitative methods include semi-structured interviews with eight caregivers, exploring usability, perceived benefits, and barriers to adoption.

**Results:**

Data will be analyzed using descriptive statistics, repeated measures ANOVA, and thematic content analysis for qualitative data. Findings will inform future studies on integrating wearable assistive technologies into caregiving workflows.

**Discussion:**

This study will provide essential insights into the feasibility and usability of exoskeletons in long-term care, potentially contributing to improved ergonomic conditions and caregiver well-being.

## Background

Lower back pain (LBP) is among the most common work-related musculoskeletal disorders (MSDs) affecting healthcare professionals, particularly those in long-term care settings (1). This occupational health issue is exacerbated by physically demanding tasks such as repositioning patients, lifting, and assisting in daily activities, which place significant strain on the lumbar spine (2). A systematic review identified a direct causal relationship between caregiving activities, such as dressing and bathing patients, and lower lumbar pain (2). Additionally, studies indicate that standing and walking without patient interaction, as well as bending, reaching, and turning, contribute significantly to cumulative lumbar compression among caregivers (3).

**Figure 1:**
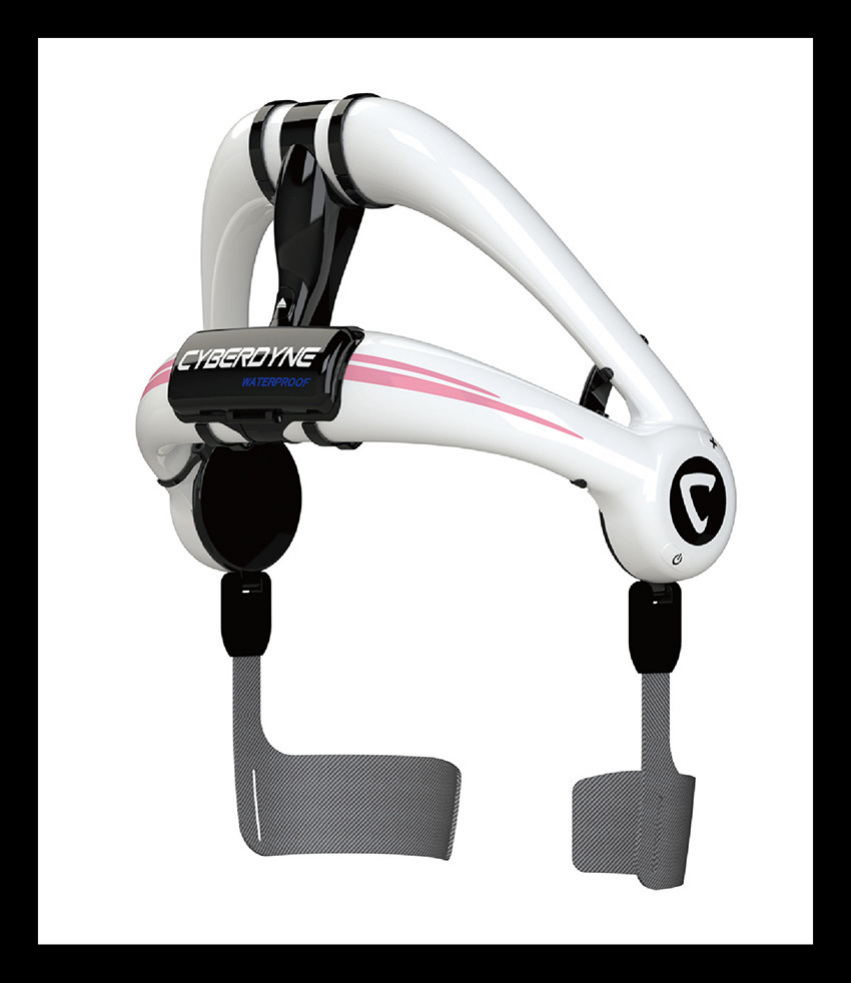
The HAL® Lumbar Type exoskeleton (CYBERDYNE Inc., Japan), shown here in its waterproof version. The device supports the lower back during physically demanding tasks and features an active control system that utilizes bioelectrical signals to assist movement.

Despite ongoing efforts to implement ergonomic workplace interventions, existing strategies, such as transcutaneous electrical nerve stimulation (1) and physiotherapy-based movement exercises (4), have shown limited efficacy in addressing LBP in caregivers. Recent findings suggest that Digital Assistive Technologies (DAT), such as wearable exoskeletons, may offer a novel approach to reducing physical strain and supporting healthcare professionals in their daily tasks (5). In recent years, lumbar exoskeletons have increasingly been introduced into industrial and manufacturing settings to mitigate the physical strain associated with manual labor tasks. A systematic review by Cardoso et al. (6) highlights the growing number of studies on passive and active exoskeletons for workplace use, particularly focusing on their potential to reduce musculoskeletal strain and support physical performance in lifting, bending, and overhead work situations. While much of the existing research has been conducted in laboratory environments, the review calls for more real-world evaluations, especially in underexplored sectors like healthcare and long-term care. While the use of occupational exoskeletons has been extensively explored in domains such as manufacturing and construction, their implementation in healthcare remains in its nascent stages. A study by Scheer et al. (7) specifically examined the social contexts of exoskeleton implementation among nurses and nurse managers with musculoskeletal disorders in long-term care. The findings underscore the significance of incorporating user concerns, including perceived stigma, workplace culture, and organizational support, to facilitate successful adoption. In comparison with numerous commercially available passive exoskeletons, the HAL® Lumbar Type exoskeleton is distinguished by its active control system, which is based on electromyographic signal detection (Figure 1). This capability enables the device to respond to the user's motor intent, potentially offering a more adaptive and user-centered form of support during caregiving activities. Furthermore, as Chao et al. (8) contend, active systems have the potential to expand access to physically demanding occupations by diminishing barriers for older workers and women. This, in turn, could contribute to the realization of inclusion and diversity objectives within the workforce. Preliminary research indicates that caregivers are amenable to utilizing exoskeletons. These devices offer the potential to mitigate work-related injuries, enhance physical well-being, and optimize work performance (9). However, despite the promising potential of technical advancements, recent research underscores a critical gap between the documented ergonomic benefits of exoskeletons and their practical implementation in real-world work environments. Lefint and Moniz (10) designate this phenomenon as the “*dilemma behind the product*,” thereby underscoring the notion that a considerable number of devices remain underutilized due to inadequate user engagement, an absence of social acceptance, and an insufficient degree of congruence with quotidian work routines.

**Figure 2:**
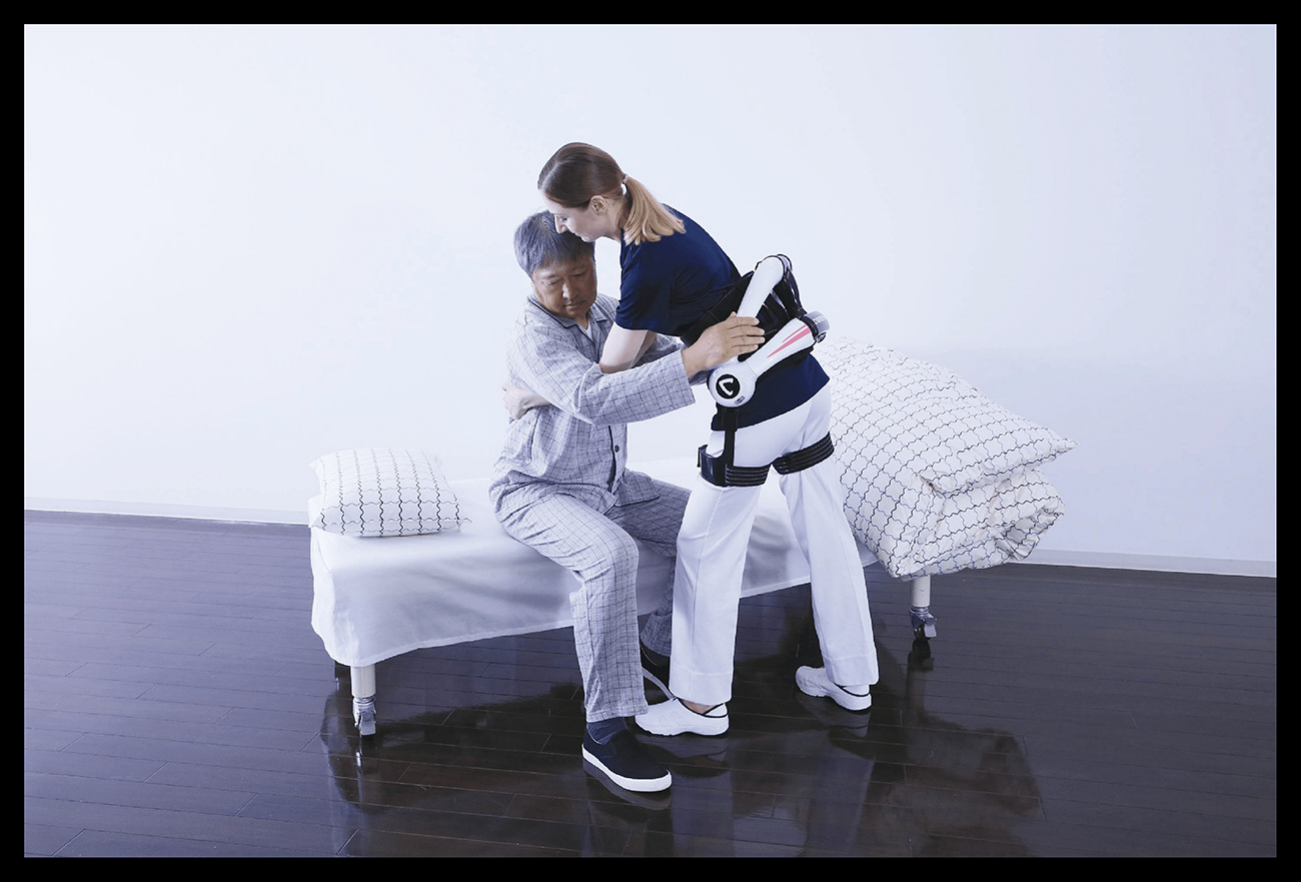
Practical application of the HAL® Lumbar Type Exoskeleton (HAL-LB03) during a patient transfer scenario. The device supports the caregiver's lower back while performing physically demanding tasks in a care setting. Reprinted with permission from HAL® Lumbar Type Exoskeleton product materials by Cyberdyne Inc., all rights reserved, https://www.cyberdyne.jp.

**Figure 3:**
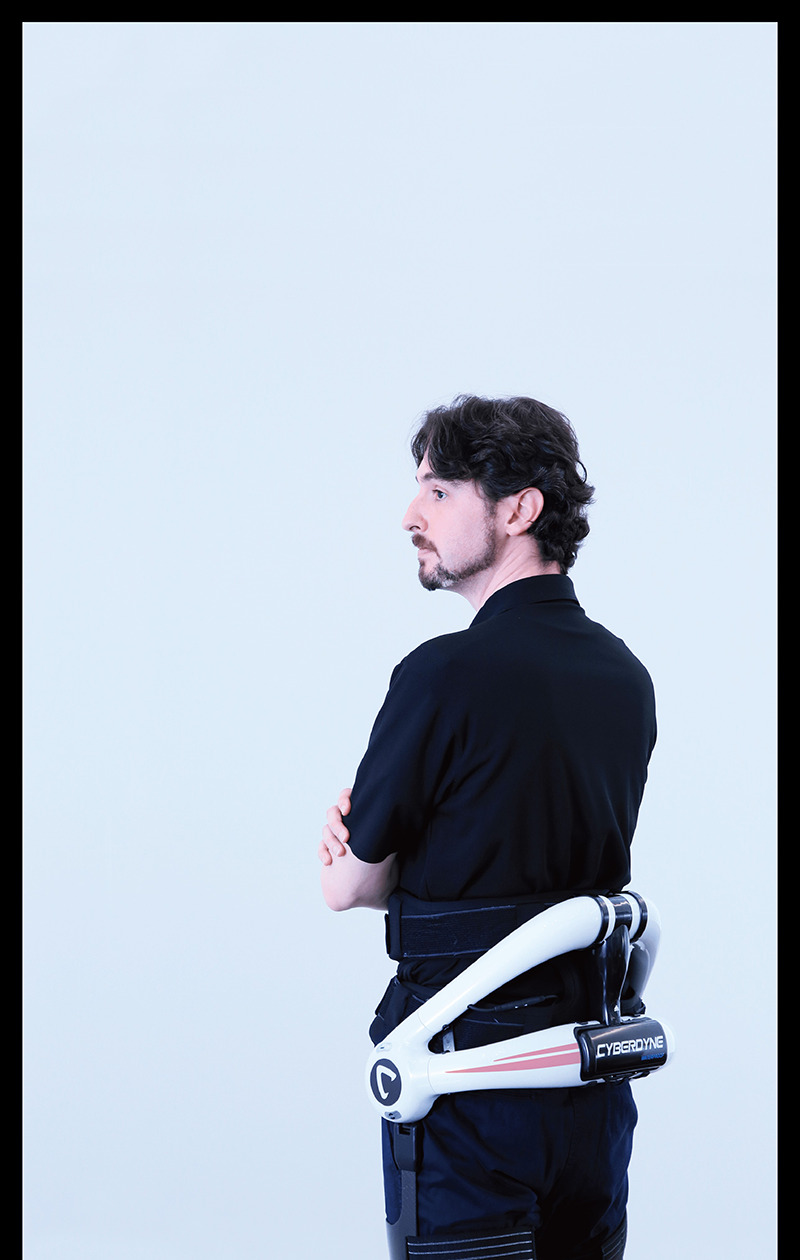
Rear view of a caregiver wearing the HAL® Lumbar Type Exoskeleton. The device is designed to reduce physical strain on the lower back during physically demanding care tasks, such as patient handling. Reprinted with permission from HAL® Lumbar Type Exoskeleton product materials by Cyberdyne Inc., all rights reserved, https://www.cyberdyne.jp.

The HAL® Lumbar Type Exoskeleton (HAL-LB03), developed by Cyberdyne Inc., is an active, battery-powered exoskeleton designed to assist the lower back during physically demanding tasks (5) (Figure 2). The device utilizes surface electromyography (EMG) to detect the wearer's bioelectrical signals (BES), subsequently translating these signals into assistive motion. This functionality aims to support natural movement patterns while mitigating strain on the lumbar spine. The system, with a weight of approximately 3.1 kilograms, including the battery, is characterized by its compact and lightweight design, which facilitates prolonged use without causing significant fatigue. The device offers five adjustable levels of support, a battery life of approximately 4.5 hours, and can be fully charged in about 2 hours (Figure 3). The device has been certified in accordance with ISO13482 (safety standard for personal care robots) and the European Machinery Directive. It is protected against dust (IP5X) and splashing water (IPX4), allowing for safe use in various care settings (5). Early studies indicate that exoskeletons have the potential to reduce lumbar strain during patient transfers, yet scientific evidence regarding their usability, potential impact, and feasibility in real-world long-term care settings remains scarce (11).

### Rationale for the Study

To date, no comprehensive studies have evaluated the real-world application of the HAL® Lumbar Type Exoskeleton in long-term care. While preliminary findings suggest that exoskeletons may improve caregiver well-being by reducing perceived physical workload, their integration into everyday caregiving practices remains underexplored (11, 12). Prior research has primarily focused on simulated care environments rather than actual clinical workflows, making it unclear whether exoskeleton-assisted care provides a tangible ergonomic advantage over existing techniques (13).

Furthermore, exoskeleton implementation in healthcare requires a multidisciplinary approach, incorporating perspectives from caregivers, healthcare managers, and patient safety specialists (14). Research on user acceptance and the practical integration of exoskeletons into caregiving routines remains limited. The lack of data on potential adoption barriers, ergonomic benefits, and long-term usability presents a critical knowledge gap that this study aims to address.

### Study Objectives

This study aims to evaluate the feasibility, usability, and user acceptance of the HAL® Lumbar Type Exoskeleton in long-term care settings by addressing the following research questions:

1. Feasibility—Can the HAL®lumbar be effectively integrated into daily caregiving workflows without disrupting patient care?
2. Usability—How do caregivers perceive the device in terms of comfort, mobility, and ease of use?
3. Mode of Action—Is there a perceived reduction in self-reported lower back pain among caregivers?
4. User Acceptance—To what extent are caregivers willing to adopt the exoskeleton as a long-term assistive tool in their work environment?

By applying a mixed-methods research design, this study will assess the real-world impact of the HAL® Lumbar Type Exoskeleton in long-term care, providing empirical data on its practical integration, perceived usefulness, and potential to reduce physical strain. These findings will inform future large-scale trials and contribute to the broader discussion on DAT in healthcare. This study follows a sequential explanatory mixed-methods design, consisting of a quantitative phase followed by a qualitative phase. The study design is non-randomized, exploratory, and intervention-based, evaluating the feasibility, usability, and perceived benefits of the HAL® Lumbar Type Exoskeleton among caregivers in long-term care facilities.

## Methods

### Study design

This study is a prospective, non-randomized, exploratory interventional feasibility study conducted in three long-term care facilities. It applies a sequential explanatory mixed-methods design, beginning with a quantitative phase to collect structured data, followed by a qualitative phase to contextualize and deepen understanding of the results. The study integrates both quantitative and qualitative data to evaluate the feasibility, usability, and perceived benefits of the HAL® Lumbar Type Exoskeleton in real-world caregiving environments. According to the MRC Framework for Complex Interventions, the study falls within the “Feasibility and Piloting” phase (15).

**Figure 4:**
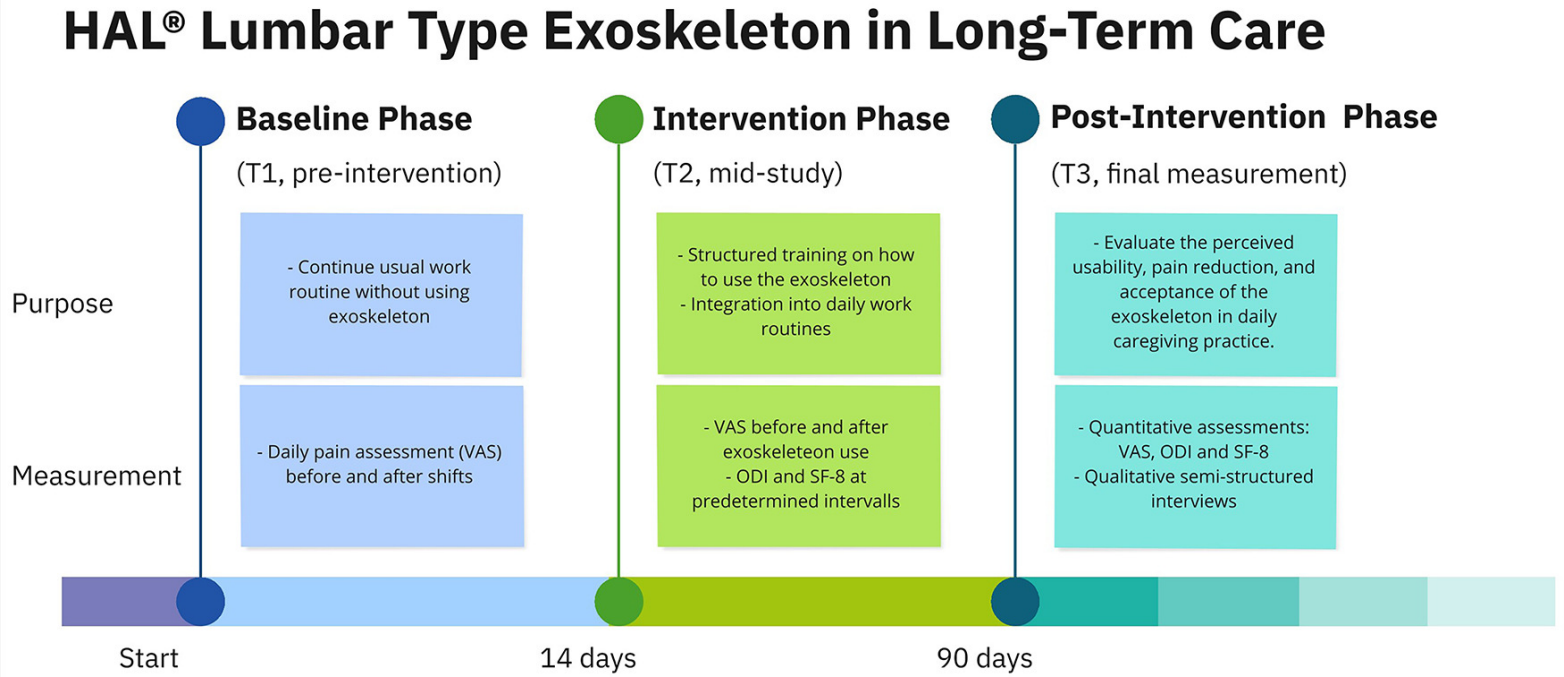
Methodological timeline of the exoskeleton study, illustrating the three study phases (Baseline, Intervention, Post-Intervention) and corresponding measurement time points (T1–T3). The figure outlines the temporal structure, content, and assessment tools used to evaluate the HAL® lumbar exoskeleton in daily caregiving practice. Reprinted with permission from HAL® Lumbar Type Exoskeleton product materials by Cyberdyne Inc., all rights reserved, https://www.cyberdyne.jp.

The study is divided into three main phases (Figure 4):

1. Baseline Phase (T1, pre-intervention): A 14-day period in which caregivers continue their usual work routine without using the exoskeleton, while daily pain levels are recorded using the (VAS). This phase aims to establish an individual baseline for back pain intensity.
2. Intervention Phase (T2, mid-study measurement): A 90-day period during which caregivers are expected to integrate the HAL® Lumbar Type Exoskeleton into their daily work routines. During this period, the collection of data is ongoing. Pain levels are recorded using the VAS before and after exoskeleton use on selected days. Additionally, the Oswestry Disability Index (ODI) and the Short-Form Health Survey (SF-8) are administered at predetermined intervals.
3. Post-Intervention Phase (T3, final measurement): Upon the culmination of the intervention period, comprehensive quantitative assessments and in-depth qualitative interviews will be conducted to evaluate the perceived usability, pain reduction, and acceptance of the exoskeleton in daily caregiving practice.

### Participants and recruitment

This study's recruitment process targeted caregivers employed in long-term care facilities, seeking to participate in a feasibility trial assessing the HAL® Lumbar Type Exoskeleton. The sampling strategy employed a snowball sampling approach (16), targeting institutions with prior collaborative involvement in related research endeavors. The study's objectives and procedures were communicated to long-term care facility managers, who subsequently approached their caregiving staff to ascertain their interest in participating. However, to ensure a balanced and participatory decision-making process, the final decision to conduct the study in a given facility was made in collaboration with caregivers themselves, rather than being dictated solely by facility management. To facilitate this shared decision-making approach, the research team visited each potential study site in person. During these visits, caregivers received detailed study briefings, had the opportunity to ask questions, and were encouraged to express their willingness or concerns regarding participation. The initial visits were designed to ascertain the caregivers’ preliminary interest in participating, and did not include the option to test the exoskeleton, in order to avoid undue influence from external factors. This approach was adopted to ensure that participation was voluntary and based on informed consent, rather than being determined solely by management directives. Following these consultations, three facilities were selected as study sites. The implementation of this strategy was driven by the need to mitigate potential bias toward technology-affine institutions and to ensure that the study population was representative of real-world caregivers across a range of technology openness and workplace conditions. However, it must be acknowledged that the implementation of a snowball sampling approach may have resulted in the introduction of a selection bias, potentially favoring institutions that have demonstrated prior research involvement or a more proactive stance toward innovation. Additionally, there is a possibility that there is an overrepresentation of caregivers who are motivated on an individual basis or who are technologically open, despite the efforts to include diverse perspectives. These limitations will be considered in the interpretation of the study findings.

### Inclusion criteria

Participants were eligible for inclusion if they met the following criteria:

• At least 18 years of age.
• Employed as a caregiver in a participating long-term care facility.
• Regularly engaged in physically demanding caregiving tasks involving patient transfers, repositioning, and mobility assistance.
• Existing back pain
• Ability and willingness to wear the exoskeleton for a three-month period during work shifts.
• Presence of clinically diagnosed lower back pain that had been perceived as problematic for at least three months and was aggravated by work-related physical demands.

### Exclusion criteria

Participants were excluded from the study if they met any of the following criteria:

• Severe musculoskeletal disorders that would prevent the safe use of the exoskeleton.
• Current pregnancy, to minimize any potential risks.
• Ongoing medical conditions or treatments that could interfere with safe participation (e.g., recent surgery, ongoing rehabilitation therapy).

This rigorous recruitment process, emphasizing voluntary participation and shared decision-making, ensured a representative and engaged study population while minimizing potential selection biases.

### On-Site implementation

The intervention is conducted directly at the study sites to ensure real-world applicability. Before the start of exoskeleton use, all participants attend training sessions, which include both theoretical instruction and practical exercises (17, 18). These sessions are led by trained facilitators who guide caregivers on:

• Proper device fitting and adjustment to ensure comfort and functional impact.
• Optimized movement patterns to maximize support while reducing strain.
• Technical familiarization with the system's functionalities. Handling of potential technical challenges that might arise during daily use.

Caregivers are required to don the exoskeleton during their assigned work shifts, particularly during periods of elevated workload, such as the morning hours between 6:00 a.m. and 10:00 a.m., as well as the late shift between 6:00 p.m. and 10:00 p.m., with an emphasis on tasks such as:

• Patient lifting and repositioning.
• Assisting with mobility and transfers (e.g., bed-to-chair transitions).
• General physical activities that typically contribute to lower back strain​ (putting on compression stockings to prevent thrombosis).

### Primary and secondary outcome measures

The present study is of an evaluative nature, with the objective of determining the impact of the HAL® Lumbar Type Exoskeleton on lower back pain in long-term care nurses. The primary outcome measure is the change in subjective pain intensity in the lower back, which is assessed using the Visual Analog Scale (VAS) at multiple time points before, during, and after the intervention. The VAS is a validated instrument that ranges from 0 (no pain) to 10 (worst imaginable pain). In this study, scores between 4 and 6 will be considered indicative of moderate/problematic pain, while scores of 7 or higher will be interpreted as severe pain, based on established clinical cut-off values (19).

Secondary outcome measures include the assessment of functional limitations using the Oswestry Disability Index (ODI) and the health-related quality of life measured by the Short-Form-Health-Survey (SF-8). Additionally, qualitative interviews will explore the perceived usability and acceptance of the exoskeleton in the caregiving process.:

### Primary outcome

• Reduction in self-reported lower back pain [measured through daily VAS assessments (20, 21) before and after work shifts].

### Secondary outcomes

• Functional improvement measured via ODI (22) at three time points (T1, T2, T3).
• Changes in health-related quality of life assessed through SF-8 (23, 24) surveys.
• User acceptance and feasibility explored through qualitative interviews with selected participants.

### Data collection

The intervention involves the integration of the HAL® Lumbar Type Exoskeleton into the daily workflow of caregivers in long-term care facilities. The objective of the study is to evaluate the feasibility, usability, and potential impact of this exoskeletal support in reducing lower back strain among healthcare professionals. The study intervention consists of three key phases:

1. Baseline Measurement Phase (T1, Pre-Intervention, 14 Days)
2. Prior to using the exoskeleton, caregivers continue their daily work routine without the device for a period of 14 days.
3. Daily pain assessment using the VAS before and after shifts to establish an individual baseline without exoskeleton use.
4. The purpose of this baseline phase is to establish a comparative reference value for back pain, which allows an assessment of pre-existing pain levels and its potential modification following exoskeleton use
5. Implementation of the Exoskeleton (T2, 90-Day Intervention Phase)
  • After the baseline assessment, participants receive structured training on how to use the HAL® Lumbar Type Exoskeleton effectively in their work environment and are then instructed to use the exoskeleton during their physically demanding work periods (e.g., morning and evening shifts).
  • Daily pain assessments VAS continues, allowing comparison before and after work shifts to determine pain reduction.
6. Additional functional assessments include the ODI and the SF-8, collected at mid-study (T2).
7. Post-Intervention Evaluation (T3, Final Measurement)
  • At the end of the 90-day intervention, a final round of quantitative assessments is conducted:
    • VAS (pain measurement before and after work shifts).
    • ODI and SF-8 surveys to assess functional improvements and overall well-being.
  • Qualitative semi-structured interviews are conducted with a subset of eight caregivers to gain in-depth insights into their experiences with the exoskeleton.
  • These interviews explore:
    • Usability and comfort perceptions.
    • Potential ergonomic and workflow improvements.
    • Barriers and facilitators of exoskeleton adoption in routine care.

Quantitative data:

• VAS for pain assessment (baseline and measured pre- and post-use) (20, 21).
• ODI for functional disability (22).
• SF-8 for general health-related quality of life (23, 24).

All quantitative data are recorded using structured paper-based forms, which are subsequently transferred into SPSS for statistical analysis.

Qualitative data:

• Semi-structured interviews with eight caregivers focusing on usability and comfort perceptions of the exoskeleton, barriers and facilitators for integration into daily work routines, long-term feasibility and adoption potential (25, 26).

The interviews are conducted on-site in the participating care facilities and are audio-recorded, transcribed verbatim, and analyzed using qualitative content analysis. The Systematic Text Condensation (STC) method by Malterud (2 1) is applied, which involves:

1. Familiarization with the entire dataset to identify overarching themes.
2. Extraction of meaningful units related to usability, feasibility, and experience with the exoskeleton.
3. Condensation and categorization of extracted statements.
4. Synthesis of findings into coherent thematic descriptions.

Two researchers will independently code the qualitative data to ensure inter-rater reliability; discrepancies will be resolved by consensus.

### Analysis and data triangulation

The present study utilizes a sequential explanatory mixed-methods design (27), comprising a quantitative phase, followed by a qualitative phase. The integration of both datasets is imperative to derive comprehensive insights into the usability, feasibility, and impact of the HAL® Lumbar Type Exoskeleton in long-term care settings.

The statistical analysis of the quantitative data employs a descriptive and inferential approach to evaluate the feasibility of the HAL® Lumbar Type Exoskeleton in reducing lower back pain and improving the functional capacity of caregivers. Descriptive statistics, including means, standard deviations, and frequency distributions, will be used to summarize the characteristics of the study population and key outcome variables. To assess changes in pain perception (VAS scores) over time, a repeated measures ANOVA will be conducted to compare mean values across the three study phases (T1, T2, and T3). For the ODI and SF-8, pre-post comparisons will be conducted using paired-sample t-tests or non-parametric alternatives, depending on data distribution. For all inferential tests, a significance level of *p* < 0.05 will be employed. The objective of the present analyses is to provide insight into the potential contributions of the exoskeleton to pain reduction and improvements in caregivers’ perceived physical well-being and functional capacity (28).

A key methodological aspect of this study is the true integration of quantitative and qualitative data within a mixed-methods framework (27). Rather than analyzing both datasets in isolation, the qualitative results will serve to contextualize and explain the quantitative findings. This sequential explanatory mixed-methods approach follows the quantitative results guiding the thematic analysis of qualitative data (27). For instance, if statistical analyses indicate a significant reduction in pain levels (VAS), the qualitative data will be examined to explore how caregivers subjectively experience these changes and whether they attribute them to the exoskeleton. In a similar vein, findings from structured surveys on caregivers’ experiences with the exoskeleton will be compared with interview data to assess alignment between reported perceptions of comfort, ease of use, and potential barriers to adoption. This approach ensures that statistical results are not interpreted in isolation but rather enriched through qualitative perspectives. The final stage of analysis involves an interpretation-level integration, in which patterns from both datasets are mapped onto each other to develop a comprehensive understanding of the feasibility and usability of exoskeleton-assisted caregiving. This integration enables a more holistic evaluation, ensuring that both objective measures and subjective experiences contribute to the study's overall conclusions.

## Ethics approval

All procedures involving human participants or human tissue will be performed in accordance with the institutional and national research committee ethical standards and tenets of the 1975 Declaration of Helsinki (29) and its later amendments or comparable ethical standards. Informed consent will be obtained from all the participants. This study was approved by the Ethics Committee of the Faculty of Medicine, Martin-Luther-University Halle-Wittenberg (approval no. 2024-114, dated February 20, 2025). The study was registered in the German Register of Clinical Studies (registration no. DRKS00036285), and the protocol has not been published previously.

## Results

### Expected findings

The objective of this study is to generate empirical insights into the feasibility, usability, and potential impact of the HAL® Lumbar Type Exoskeleton in long-term care settings. The primary outcome of the study is the change in self-reported lower back pain intensity among caregivers using the exoskeleton, measured via the (VAS). The investigation will examine whether the use of the exoskeleton leads to a measurable reduction in pain levels over the course of the intervention. To evaluate this, pain intensity will be meticulously monitored on a daily basis over a 14-day baseline phase (T1) and subsequently contrasted with pain levels during (T2) and after the intervention phase (T3). In addition to the pain reduction, the secondary outcomes focus on the functional impact of the exoskeleton on caregivers’ daily work. The ODI is used to evaluate the degree of physical limitations in caregiving tasks, and the SF-8 is used to assess caregivers’ overall well-being and quality of life. These assessments will provide insights into whether the exoskeleton supports caregivers by reducing physical strain and enhancing work-related health outcomes. Specifically, we anticipate the following key results:

• Reduction in self-reported back pain (VAS) after using the exoskeleton.
• Improved functional mobility and reduced strain (ODI).
• Enhanced usability ratings and willingness to integrate exoskeletons into routine care.
• Identification of barriers and facilitators to adoption in long-term care.

Another key research question concerns the actual supportive function of the HAL® exoskeleton. While previous studies have suggested that exoskeletal assistance may alleviate musculoskeletal strain, this study will determine whether these theoretical benefits translate into practical advantages in real caregiving environments. To this end, qualitative semi-structured interviews will be conducted with a selected group of caregivers at the conclusion of the study. These interviews will explore their subjective experiences, challenges, and perceived usability of the exoskeleton.

To ensure a comprehensive interpretation, this study employs a sequential explanatory mixed-methods approach, in which quantitative findings are complemented by qualitative insights. The statistical results from the VAS, ODI, and SF-8 assessments will provide an objective measure of pain reduction and the impact on caregivers’ physical health and well-being. Meanwhile, the qualitative interviews will contextualize these results by exploring user experiences, acceptance, usability perceptions, and potential barriers to adoption. For instance, if the quantitative analysis reveals a substantial decrease in pain scores, the qualitative data will elucidate how caregivers interpret this change, whether they ascribe it directly to the exoskeleton, and the factors that influenced their experience. Conversely, if the quantitative results demonstrate limited impact, the qualitative insights will identify potential barriers, such as discomfort, mobility restrictions, or reluctance to use the device consistently. The integration of numerical trends with caregiver narratives is a methodological approach that ensures a comprehensive evaluation of the exoskeleton's feasibility and usability in long-term care settings. The findings of this study will inform future research, support potential modifications to exoskeletal technology, and provide practical recommendations for its implementation in healthcare environments.

## Discussion

The objective of this study is to generate insights into the usability, feasibility, and potential impact of the HAL® Lumbar Type Exoskeleton in long-term care settings. As the study is still ongoing, no final results can yet be presented. However, based on previous research and the structure of this study, several principal results can be expected.

### Principal results

It is anticipated that the utilization of an exoskeleton will lead to a decrease in self-reported lower back pain among caregivers, as indicated by the VAS. This phenomenon is expected to be more evident among participants who frequently utilize the device during physically demanding caregiving activities. Additionally, an improvement in functional ability and overall well-being is expected, as reflected in changes in ODI and SF-8 scores. The qualitative component of the study is expected to provide deeper insights into user experiences, highlighting both facilitators and barriers to the integration of exoskeletal technology in daily care routines. Given prior findings on assistive technologies in healthcare, it is likely that acceptance and perceived usability will vary among caregivers, with some individuals experiencing initial skepticism or discomfort, while others may perceive significant ergonomic benefits.

### Limitations

Despite its strengths, the study has several limitations that must be acknowledged. First, the relatively small sample size may limit the generalizability of findings to broader populations of caregivers. Additionally, as the study is conducted in real-world care settings, factors such as variability in work routines, differences in staff training, and facility-specific conditions may introduce heterogeneity in the results. Another limitation concerns user adherence. While participants are instructed to wear the exoskeleton during physically demanding tasks, actual usage patterns may differ from the intended implementation, potentially resulting in variability in exposure to the intervention. Furthermore, the long-term effects of the intervention beyond the three-month study period remain unknown, necessitating future follow-up studies to assess the sustainability of potential benefits.

The study's restriction to long-term residential care settings may limit the generalizability of results to other care environments, such as home care or ambulatory nursing services, where caregivers have shorter patient visits, frequent transitions between locations, and potential constraints related to driving and transport logistics. The need for frequent donning and doffing of the exoskeleton may pose practical challenges, potentially limiting its feasibility in such settings. Future research should explore how exoskeletons can be adapted to diverse healthcare environments to ensure broader applicability.

### Comparison with prior work

In contrast to prior research on exoskeletal support in physically demanding professions, this study offers a distinctive contribution by focusing specifically on long-term care professionals. While exoskeletons have been investigated in industrial and rehabilitation settings, evidence on their impact and feasibility in healthcare environments remains limited. Most prior studies have primarily examined kinematic and biomechanical effects (30) under theoretical conditions (11, 12). In contrast, this study adopts a mixed-methods approach, integrating quantitative pain and function assessments with qualitative user experiences. This comprehensive approach enables a more nuanced evaluation of the influence of exoskeleton use on both objective physical outcomes and subjective perceptions of usability and feasibility.

## Conclusions

This study aims to demonstrate the feasibility and usability of exoskeleton-assisted care and its potential to reduce caregiver burden. Future research should explore long-term adoption strategies and cost-effectiveness analyses. It provides important insights into the usability, feasibility, and potential health benefits of exoskeleton-assisted caregiving. By using a rigorous mixed methods approach, the study aims to bridge the gap between technical performance evaluations and practical usability considerations in healthcare settings. Future research should build on these findings by exploring long-term adherence, potential refinements to the exoskeleton design, and the broader implications of assistive technology adoption in the healthcare professions, particularly with the exoskeleton evaluated through a randomized controlled trial design. If proven effective, exoskeletons could become an integral tool for reducing caregiver strain, improving workplace ergonomics, and ultimately improving the quality of care in long-term care environments.

## Data Availability

All data will be available upon reasonable request.
